# Association between protein-to-energy ratio and overweight/obesity in children and adolescents in the United States: a cross-sectional study based on NHANES

**DOI:** 10.3389/fped.2024.1383602

**Published:** 2024-06-25

**Authors:** Feng Zhao, Yudan Wang, Zhaoyi Liu, Jiao Wang, Yinyin Xia, Xuejun Jiang, Lixiao Zhou, Ahmad Khan, Shuqun Cheng, Zhen Zou, Chengzhi Chen, Jingfu Qiu

**Affiliations:** ^1^ Department of Health Laboratory Technology, School of Public Health, Chongqing Medical University, Chongqing, China; ^2^ Research Center for Environment and Human Health, School of Public Health, Chongqing Medical University, Chongqing, China; ^3^ Department of Obstetrics and Gynaecology, The Second Affiliated Hospital of Chongqing Medical University, Chongqing, China; ^4^ Department of Occupational and Environmental Health, School of Public Health, Chongqing Medical University, Chongqing, China; ^5^ Center of Experimental Teaching for Public Health, Experimental Teaching and Management Center, Chongqing Medical University, Chongqing, China; ^6^ Molecular Biology Laboratory of Respiratory Diseases, Institute of Life Sciences, Chongqing Medical University, Chongqing, China

**Keywords:** dietary protein, overweight/obesity, children, adolescents, NHANES

## Abstract

**Background:**

The dietary protein proportion may be crucial in triggering overweight and obesity among children and adolescents.

**Methods:**

Cross-sectional data from 4,336 children and adolescents who participated in the National Health and Nutrition Survey (NHANES) between 2011 and March 2020 were analyzed. Multivariate logistic regression was used to calculate odds ratio (OR) and 95% confidence interval (CI). Restricted cubic splines assessed the nonlinear relationships between dietary protein intake and the prevalence of overweight and obesity.

**Results:**

Adjusted logistic regression models showed that each 1% increase in dietary protein proportion was associated with a 4% higher risk of overweight and obesity (OR = 1.04, 95% CI: 1.01–1.07). A nonlinear relationship was noted in children aged 6–11 years (*P* < 0.05), as demonstrated by restricted cubic spline analysis. After dividing dietary protein intake into quartiles, the highest quartile had an adjusted OR of 2.07 (95% CI: 1.35, 3.16, *P* = 0.001) compared to the lowest, among children aged 6–11 years.

**Conclusion:**

Dietary protein intake is positively linked to overweight and obesity in American children, irrespective of individual characteristics and total energy consumption.

## 1 Introduction

Over the past four decades, the prevalence of overweight and obesity in children and adolescents has increased alarmingly, posing a significant public health challenge (1). The World Obesity Federation's 2019 report projected that by 2025, there would be 206 million obese children and adolescents aged 5 to 19 (2). In 2016, over 340 million children and adolescents globally were overweight or obese. Specifically, in the United States, 18.5% of children aged 2–19 are obesity, and 5.2% are severely obesity (3). Current trends indicate a rising prevalence of obesity in young children (4). Obesity is now recognized as a multifaceted disease that impairs various systems and can severely affect a child's intellectual, behavioral, psychological, and sexual development, leading to potential lifelong consequences (5, 6). Moreover, overweight and obesity in youth are significantly linked with an increased risk of chronic noncommunicable diseases such as diabetes, hypertension, ischemic heart disease, and stroke in later life (7). Identifying risk factors associated with overweight and obesity during childhood and adolescence is therefore critical. A large-scale study involving 2.3 million adolescents indicated that even without reaching overweight or obesity, an increase of Body Mass Index (BMI) in adolescents aged 16–19 years is closely associated with the risk of cardiovascular disease (CVD) mortality in adulthood (8). This suggests that greater emphasis should be placed on researching and identifying risk factors related to elevated BMI in children and adolescents. Contemporary obesity treatments include dietary therapy, increased physical activity, pharmacological interventions, and bariatric surgery (9–11).

The causes of obesity in children and adolescents involves a complex interplay of genetic predisposition, environmental factors, and lifestyle choices (12, 13). Contemporary obesity treatments encompass dietary therapy, increased physical activity, pharmacological interventions, and bariatric surgery (9–11). Notably, dietary therapy has garnered increasing focus. The fourth round of WHO's European Childhood Obesity Surveillance Initiative emphasized that healthy dietary practices in childhood can prevent malnutrition and noncommunicable diseases, thereby mitigating overweight and obesity rates (14). Daniela Ner et al. identified ultra-processed food consumption as a potential determinant of overweight and obesity in this demographic of children and adolescents (15). Moreover, diet not only influences body weight but also contributes to the etiology of other chronic diseases such as heart disease, stroke, and type 2 diabetes (16). Evidence suggests that the proportion of dietary protein significantly impacts the development of obesity in children and adolescents (17). Previous research indicated that diets high in protein might promote greater weight and fat loss (18). However, epidemiological studies have shown that children with obesity tend to consume more protein than their healthy-weight counterparts (19–21). In summary, the research examining the link between dietary protein intake and obesity among children and adolescents is currently contentious.

This study utilized data from the National Health and Nutrition Survey (NHANES) to examine the association between dietary protein intake and the risk of overweight/obesity in American children and adolescents. We hypothesized that higher protein intake might constitute a potential risk factor for overweight/obesity in this group. Our goal is to provide a scientifically robust foundation to inform recommendations for obesity prevention and control, and support the development of dietary guidelines for children and adolescents.

## 2 Methods

### 2.1 Study design

The present study utilized cross-sectional data from the NHANES spanning March 2011 to 2020. NHANES employs a complex, stratified multi-stage probability sampling method for data collection (22). The survey protocol has been approved by the National Center for Health Statistics' Research Ethics Review Board, and informed consent was obtained in writing from all participants. NHANES combines in-home interviews with physical examinations, including a 24-h dietary recall interview conducted in-person at Mobile Examination Centers (MECs), followed by a secondary 24-h dietary recall via telephone interviews three to ten days later. The survey's administration is overseen by the National Center for Health Statistics (NCHS) and the Centers for Disease Control and Prevention (CDC), with a comprehensive methodology description available in prior publications (23).

### 2.2 Inclusion criteria

**Figure 1:**
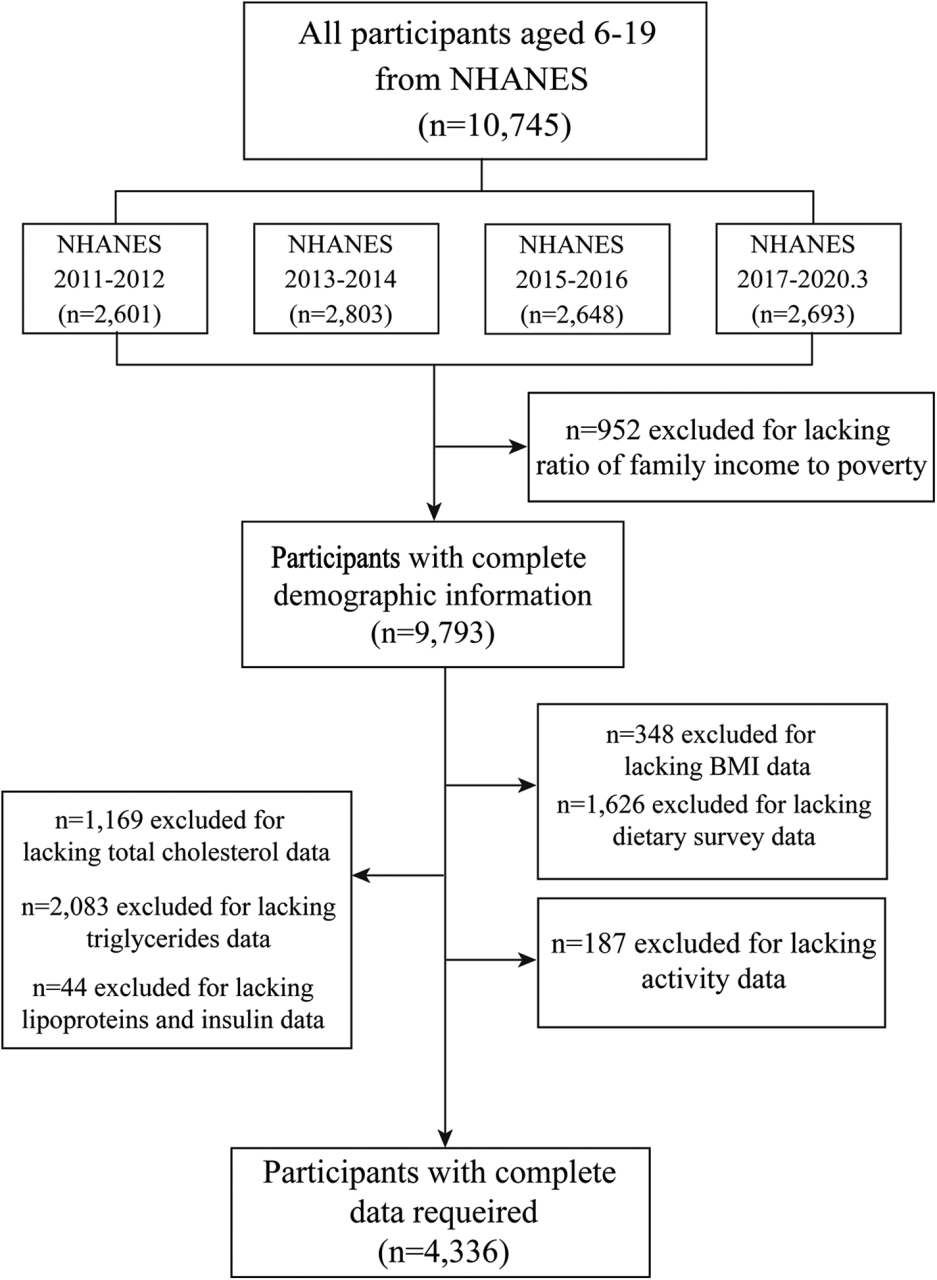
Flow chart of inclusion and exclusion criteria for the study sample. NHANES, National Health and Nutrition Survey; BMI, Body Mass Index.

The analysis included adolescents aged 6–19 years who had complete and reliable two-day dietary intake data. We excluded participants with incomplete nutritional information, lacking relevant body measurements, or missing physical activity data, resulting in a final sample size of 4,336 participants. A detailed study flowchart is depicted in Figure 1.

### 2.3 Exposure and outcome variables

NHANES collects dietary data through a 24-h dietary recall method. Assisted by a proxy when necessary, up to two 24-h dietary recalls were conducted for each child aged 6–11 and for participants aged 12 and above. The dietary recall process is administered in person initially and via telephone subsequently by a trained interviewer. Each subject or their proxy was asked to recount all foods and beverages consumed in the previous 24 h (from midnight to midnight) (24). Nutrient intake, specifically protein, carbohydrates, and fats, was calculated using the USDA's Food and Nutrient Database for Dietary Studies. We calculated the mean nutrient intake from the two 24-h dietary recalls and assessed macronutrient consumption levels as a percentage of the total energy intake (E%) (25). Additionally, NHANES utilized a multiple-pass method, which includes the collection of self-reported food inventories by participants, probing for forgotten foods, gathering details about consumed foods, and finally, exploring any additional food items (26). Simultaneously, a set of standard measurement tools (such as grids, cups, spoons, and bowls) was used to assist respondents in reporting and calculating the quantities of consumed food (27).

Participants' height and weight were measured by trained health technicians at the Mobile Screening Centre (MEC). Body mass index (BMI) was computed by dividing the weight in kilograms by the square of height in meters. Utilizing the 2000 CDC Growth Charts, NHANES classified the BMI for individuals aged 6–19 years into four categories: underweight (BMI < 5th percentile), healthy weight (5th ≤ BMI <85th percentile), overweight (85th ≤ BMI <95th percentile), and obesity (BMI ≥95th percentile) (28).

### 2.4 Covariates

Demographic data were obtained via questionnaires, capturing information on gender, age (in years), the household income-to-poverty ratio (PIR), and ethnicity (categorized as Mexican American, other Hispanic, non-Hispanic white, non-Hispanic black, non-Hispanic Asian, or other). Continuous variables such as total dietary energy intake were computed from dietary recall data. We examined dietary energy (kcal), fat intake (E%), and carbohydrate intake (E%) as potential confounders in the relationship between dietary energy and obesity.

The PIR was determined based on household income relative to the Department of Health and Human Services' poverty guidelines, with thresholds set at ≤130% and >130% (29). At the MEC, participants reported physical activity levels through a questionnaire that detailed their typical weekly activities (24). Following the criteria established by Mary et al. (29), physical activity levels are classified as: Recommended (≥150 min of moderate intensity or ≥75 min of vigorous intensity per week, or an equivalent combination); Moderate (10–149 min of moderate intensity or 5–74 min of vigorous intensity per week, or an equivalent combination); Low (below the thresholds for recommended or moderate activity).

The ratio of reported energy intake (rEI) to the predicted energy requirement (pER) was utilized to assess the accuracy of dietary reports, following the rEI:pER method established by previous researchers (19). The pER is calculated using sex, age, and weight-specific equations from the American Dietary Reference Intakes. To identify implausible rEI values, we employed the approach developed by Huang et al. (19), measuring the rEI/pER percentage. An rEI that deviated from the 78% to 122% range of the pER was considered potentially unreliable. Based on this criterion, 1,092 children and 919 adolescents were excluded in the sensitivity analysis.

### 2.5 Statistical analysis

To elucidate the complexity of survey designs, we utilized NHANES sample weights spanning 9.3 years, from 2011 to March 2020, derived from day-2 dietary sample weights. These weights adjust for differential probabilities of selection, non-response, and aspects of the survey's design (23). Descriptive statistics were calculated for the entire cohort and across strata of dietary protein consumption. The sample was divided by age into children (6–11 years) and adolescents (12–19 years). Results are presented as weighted means and standard deviation [Mean (SD)] or as weighted percentages (%) for baseline characteristics and anthropometric data, with “n” representing the raw sample size. Continuous variables were analyzed using the t-test (for normally distributed data) or the Kruskal-Wallis test (for skewed distributions). Associations between categorical variables were examined using Chi-square or Fisher's exact tests. Multiple logistic regression was employed to explore the link between dietary protein intake (treated as a continuous predictor) and overweight/obesity risk. For a nuanced assessment of this relationship, we utilized restricted cubic splines with knots at the 5th, 35th, 65th, and 95th percentiles. To address non-linearity, quartile midpoints of estimated dietary protein intake (Q4, Q3, Q2, Q1) at the 87.5th, 62.5th, 37.5th, and 12.5th percentiles were calculated. Risks at the 87.5th, 62.5th, and 37.5th percentiles were compared to those at the 12.5th percentile, using the *β* coefficient from logistic regression to estimate the adjusted OR.

Multivariate logistic regression analyses were progressively refined: Model 1 accounted for age, sex, ethnicity, and PIR; Model 2 included adjustments for total caloric intake (kcal) and dietary fiber (g) in addition to Model 1. In Model 3, we further adjusted for physical activity levels. Acknowledging that within a fixed energy framework, an elevation in protein intake must coincide with reduced intake of carbohydrates or fats, we examined the macronutrient substitution effects on the protein-obesity relationship. We utilized a Multivariate nutrient density model (30) to explore the effect on obesity/overweight when replacing protein intake with equal energy from carbohydrates (5% E) or fats (5% E) (21). Additionally, sensitivity analyses excluded participants with implausible energy intake (EI), determined by the rEI:pER method previously mentioned, to reaffirm the relationship between dietary protein intake and overweight/obesity.

All statistical analyses were conducted using R version 4.2.3. We considered a two-tailed *P*-value <0.05 as indicative of statistical significance.

## 3 Results

### 3.1 Baseline of the study population

According to the study's inclusion-exclusion criteria, an initial sample of 10,745 participants aged 6–19 years, with dietary data from the NHANES 2011 to March 2020, was considered. Exclusions applied to individuals lacking comprehensive data on household income-to-poverty ratio, height, weight, BMI, dietary intake, cholesterol, triglycerides, lipoprotein, insulin, and physical activity. Ultimately, 4,336 participants (2,194 males and 2,141 females) were included in the analysis, as shown in Figure 1.

Of the included participants, 36.4% of children and adolescents were categorized as overweight/obese, with males comprising 51.8% of this group. The prevalence of overweight/obesity was particularly high among children aged 6–11 (67.5%). Adolescents presented with higher baseline weight, height, and BMI than those in the children's group (*P *< 0.001). Dietary analysis showed that adolescents consumed less carbohydrate (*P *< 0.001) and more protein (*P *< 0.001), fat (*P *= 0.048), and fiber (*P *< 0.001) than children aged 6–11, though total energy intake did not significantly differ between the age groups (*P *= 0.172). Significant differences were also evident in physical activity levels between children and adolescents (*P *< 0.001). Table 1 details these baseline characteristics.

**Table 1:** Baseline characteristics of US children and adolescents aged 6 to 19 years, NHANES 2011–2020.

Characteristic	Age group (years)			*P_*value
	Mean (SD), median (IQR), or *n* (%)			
	Overall	6–11	12–19	
*N* ^b^	4,336	292 (67.5)	140 (32.5)	
Demographic information				
Age (Years)	10 (8–13)	9 (7–10)	15 (13–17)	<0.001
Sex				
Male	2,194 (51.8)	1,475 (52.7)	719 (50.0)	0.392
Female	2,141 (48.2)	1,452 (47.3)	690 (50.0)	
Ethnicity				
Mexican American	898 (17.1)	605 (16.7)	293 (17.8)	0.338
Other Hispanic	422 (7.5)	298 (7.9)	124 (6.6)	
Non-Hispanic White	1,222 (52.1)	810 (51.1)	412 (54.1)	
Non-Hispanic Black	1,122 (13.4)	779 (13.8)	343 (12.6)	
Non-Hispanic Asian	364 (4.3)	212 (4.2)	152 (4.6)	
Other Race	308 (5.6)	223 (6.2)	85 (4.4)	
PIR				
≤130%	1,928 (35.2)	1,341 (35.7)	587 (34.0)	0.542
>130%	2,408 (64.8)	1,586 (64.3)	822 (66.0)	
Body measure				
Weight (kg)	40.20 (29.00–57.70)	32.70 (26.00–41.40)	62.90 (51.60–77.40)	<0.001
Height (cm)	144.20 (130.90–160.00)	134.90 (126.21–144.40)	165.21 (158.20–172.70)	<0.001
BMI (kg/m^2^)	19.10 (16.40–23.00)	17.50 (15.80–20.80)	22.60 (19.60–27.00)	<0.001
BMI status^a^				
Underweight	116 (3.5)	79 (3.7)	37 (3.0)	0.582
Healthy weight	2,540 (60.1)	1,749 (59.6)	791 (61.1)	
Overweight	716 (16.0)	485 (16.8)	231 (14.5)	
Obese	955 (20.4)	614 (19.9)	341 (21.4)	
Dietary intake				
Energy (kcal)	1,831.00 (1,495.83–2,251.58)	1,846.52 (1,532.92–2,209.74)	1,775.00 (1,419.64–2,334.62)	0.172
Protein (E%)	14.30 (12.21–16.43)	14.00 (12.13–15.95)	14.73 (12.52–17.11)	<0.001
Carbohydrate (E%)	52.28 (47.70–56.77)	52.95 (48.66–57.02)	50.61 (46.05–56.31)	<0.001
Fat (E%)	34.28 (30.63–37.94)	34.00 (30.58–37.65)	35.00 (30.79–38.55)	0.048
Fiber (g)	13.15 (10.02–17.60)	12.20 (9.35–17.65)	13.65 (10.48–17.54)	<0.001
Activity (*n*, %)				
Recommended	3,640 (85.1)	2,629 (90.8)	1,011 (73.8)	<0.001
Intermediate	539 (11.9)	197 (6.4)	342 (22.9)	
Low	157 (3.0)	101 (2.8)	56 (3.3)	

PIR, income-to-poverty ratio; BMI, Body Mass Index; SD, standard deviation; IQR, interquartile range.

^a^ According to the 2000 Centers for Disease Control and Prevention Growth Chart, overweight/obesity is defined as a BMI of the same age and sex at or above the 85th percentile.

^b^ *N*, number of samples.

### 3.2 Univariate analysis of overweight/obesity among the children and adolescents

Univariate analysis, as presented in Table 2, assessed potential factors linked to overweight/obesity in children and adolescents. Significant differences in BMI were found across different ethnicities (*P *< 0.001), with Non-Hispanic White children and adolescents exhibiting the highest, and Non-Hispanic Asians the lowest, rates of overweight/obesity. Children from households with lower income-to-poverty ratios also showed a greater prevalence of overweight/obesity (*P *= 0.003). Physical activity levels varied significantly between children (*P *= 0.011) and adolescents (*P *= 0.049) across different BMI categories. Dietary factors were examined for overweight/obese individuals compared to those with healthy/low body weight. Findings indicated that overweight/obese children (*P *= 0.032) and adolescents (*P *= 0.022) consumed more fat on average. Conversely, carbohydrate intake was lower among overweight/obese individuals in both the children (*P *< 0.001) and adolescent (*P *= 0.045) groups. Moreover, overweight/obese children aged 6–11 had a significantly higher protein intake (*P *= 0.001), while overweight/obese adolescents aged 12–19 had lower dietary fiber intake (*P *= 0.008).

**Table 2:** Univariate analysis of BMI-related factors in the US population aged 6 to 19 years, NHANES 2011–2020.

	Aged 6–11 years					Aged 12–19 years				
	Mean (SD), median (IQR), or *n* (%)									
	Overall	Under/Healthy weight	Overweight^a^	Obese^a^	*P*	Overall	Under/Healthy weight	Overweight^a^	Obese^a^	*P*
*N* ^b^	2,927	1,828	485	614		1,409	828	231	341	
Male	1,475 (52.7)	932 (51.1)	237 (54.5)	306 (56.4)	0.314	719 (50.0)	429 (51.6)	112 (45.5)	172 (47.4)	0.532
Female	1,452 (47.3)	896 (48.9)	248 (45.5)	308 (43.6)		690 (50.0)	399 (48.4)	119 (54.5)	169 (52.6)	
Ethnicity										
Mexican American	605 (16.7)	314 (13.4)	123 (17.9)	168 (26.3)	<0.001	293 (17.8)	148 (13.6)	62 (24.3)	83 (26.3)	<0.001
Other Hispanic	298 (7.9)	167 (6.7)	57 (10.6)	74 (9.6)		124 (6.6)	75 (6.0)	16 (4.4)	33 (10.0)	
Non-Hispanic White	810 (51.1)	550 (54.9)	127 (48.5)	133 (41.2)		412 (54.1)	257 (61.3)	68 (44.3)	82 (38.3)	
Non-Hispanic Black	779 (13.8)	476 (13.7)	125 (14.2)	178 (14.1)		343 (12.6)	188 (10.8)	53 (15.9)	101 (16.3)	
Non-Hispanic Asian	212 (4.2)	169 (5.1)	26 (3.7)	17 (1.6)		152 (4.6)	115 (5.8)	12 (1.3)	23 (2.8)	
Other Race	223 (6.2)	152 (6.2)	27 (5.1)	44 (7.2)		85 (4.4)	45 (2.6)	20 (9.8)	19 (6.3)	
PIR										
≤130%	1,341 (35.7)	795 (32.0)	231 (40.0)	315 (43.7)	0.003	587 (34.0)	322 (31.1)	97 (38.5)	166 (40.9)	0.099
>130%	1,586 (64.3)	1,033 (68.0)	254 (60.0)	299 (56.3)		822 (66.0)	506 (68.9)	134 (61.5)	175 (59.1)	
Dietary intake										
Energy (kcal)	1,846.5 (1,532.9–2,209.7)	1,858.6 (1,534.0–2,197.0)	1,833.0 (1,555.6–2,266.2)	1,846.7 (1,514.6–2,244.1)	>0.99	1,775.0 (1,419.6–2,334.62)	1,827.4 (1,461.5–2,415.7)	1,751.8 (1,346.4–2,252.7)	1,687.2 (1,236.5–2,185.8)	0.141
Protein (E%)	14.00 (12.13–15.95)	13.82 (11.73–15.62)	14.51 (12.68–16.81)	14.27 (12.53–16.34)	0.001	14.73 (12.52–17.11)	14.75 (12.52–17.21)	15.01 (12.30–17.84)	14.67 (12.60–16.50)	0.812
Carbohydrat (E%)	52.95 (48.66–57.02)	53.58 (49.35–57.36)	51.71 (48.07–55.97)	51.53 (47.10–56.21)	<0.001	50.61 (46.05–56.31)	50.98 (46.70–56.29)	49.96 (44.76–54.92)	49.73 (44.94–56.56)	0.045
Fat (E%)	34.00 (30.58–37.65)	33.70 (30.33–37.37)	34.49 (30.65–38.33)	34.42 (31.24–38.44)	0.032	35.00 (30.79–38.55)	34.35 (30.40–37.93)	35.11 (31.30–38.23)	36.09 (31.06–40.29)	0.022
Fiber (g)	13.65 (10.48–17.54)	13.75 (10.45–17.80)	13.25 (10.80–17.26)	13.65 (10.40–16.87)	0.599	12.20 (9.35–17.65)	12.85 (9.90–18.15)	11.45 (8.05–17.60)	11.01 (8.60–5.38)	0.008
Activity										
Recommended	2,629 (90.8)	1,666 (92.0)	440 (90.8)	523 (86.7)	0.011	1,011 (73.8)	598 (75.8)	173 (77.7)	233 (64.3)	0.049
Intermediate	197 (6.4)	107 (5.2)	34 (8.0)	56 (9.2)		342 (22.9)	205 (21.8)	49 (18.8)	86 (29.5)	
Low	101 (2.8)	55 (2.8)	11 (1.2)	35 (4.2)		56 (3.3)	25 (2.4)	9 (3.6)	22 (6.2)	

BMI, Body Mass Index; US, United States; NHANES, National Health and Nutrition Survey; PIR, income-to-poverty ratio; SD, standard deviation; IQR, interquartile range.

^a^ According to the 2000 Centers for Disease Control and Prevention Growth Chart, overweight/obesity is defined as a BMI of the same age and sex at or above the 85th percentile.

^b^ *N*, number of samples.

### 3.3 The relationship between dietary protein intake and overweight/obesity

Logistic regression was utilized to elucidate the relationship between daily dietary protein intake (a continuous variable) and the prevalence of overweight/obesity in children and adolescents aged 6–19. Adjustments for relevant covariates (Model 3) revealed a marginal positive correlation between levels of dietary protein intake and the risk of overweight/obesity (OR = 1.04, 95% CI: 1.01–1.07, *P *= 0.013). Age stratification indicated that this association was significantly positive only in children aged 6–11. In the unadjusted model, each 1% increase in protein intake correlated with an 8% heightened risk of overweight/obesity for this population (OR = 1.08, 95% CI: 1.04–1.13, *P *< 0.001). This association remained robust even after controlling for confounding variables such as age, gender, ethnicity, household income relative to the poverty ratio (PIR), total energy intake, dietary fiber intake, and physical activity levels (OR = 1.08, 95% CI: 1.03–1.13, *P *< 0.001) (Table 3).

**Table 3:** Relationship between dietary protein intake (continuous variable) and overweight/obesity risk in children and adolescents after adjusting for different confounding factors.

Age (years) OR (95% CI)^d^ *P*
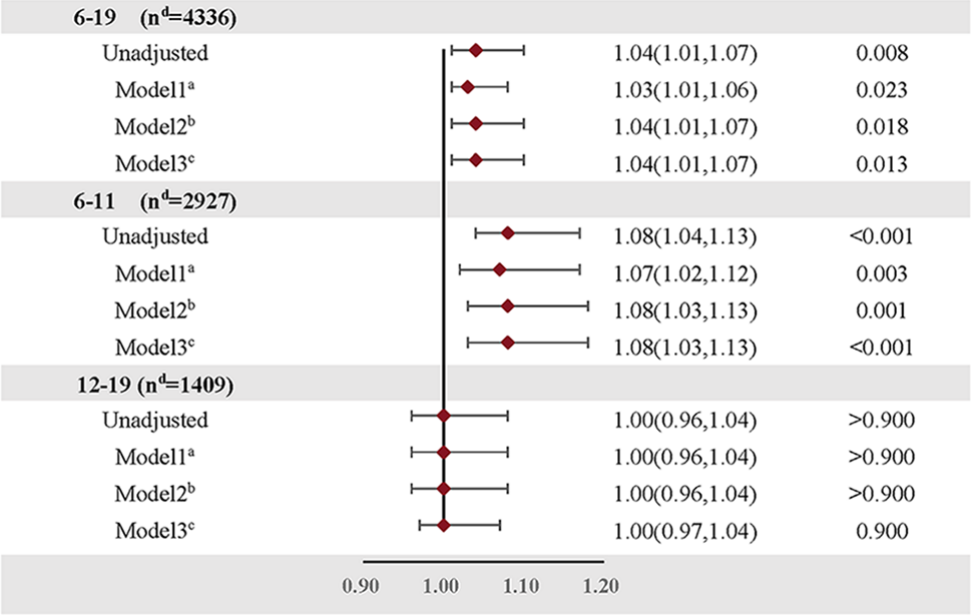

OR, odds ratio; 95% CI: 95% Confidence Interval.

^a^ Model 1, adjusted for age, gender, ethnicity and household income-to-poverty ratio (PIR).

^b^ Model 2, adjusted for covariates of model 1 plus total energy intake and dietary fiber intake.

^c^ Model 3, according to model 2 plus the physical activity level.

^d^ *n*, number of samples.

**Figure 2:**
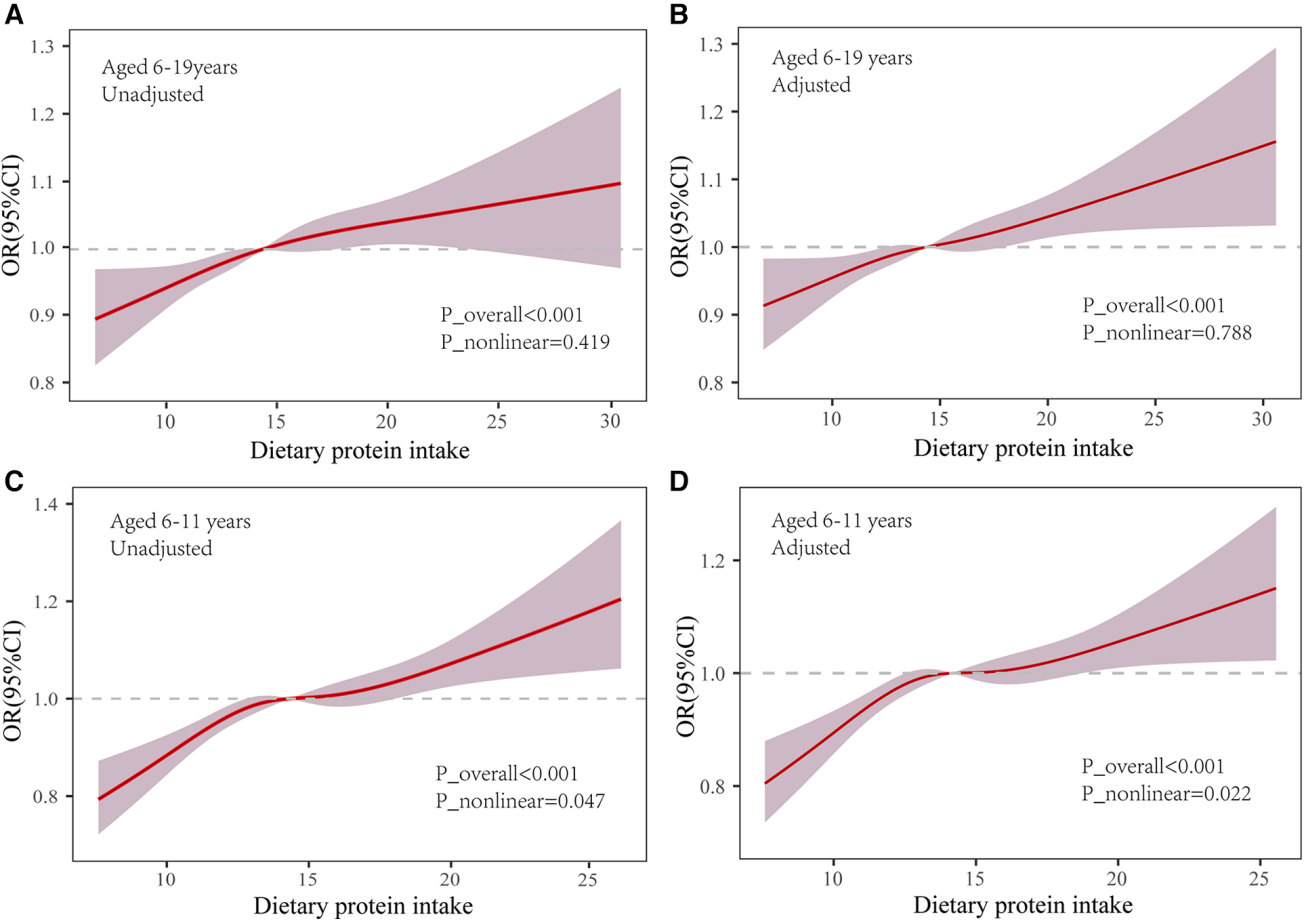
Association of dietary protein and overweight/obesity in the restricted cubic spline model. OR: odds ratio; 95% CI: 95% Confidence Interval. Use the median dietary protein intake (6–19 years: 14.92%; 6–11 years: 14.17) as reference. Adjusted P_nonlinear is the value after the adjusted age, gender, ethnicity, household income-to-poverty ratio (RIP), total energy intake, dietary fiber intake and physical activity.

Further analyses employed restricted cubic spline functions, as depicted in Figure 2, to investigate potential nonlinearity in the relationship. In the 6–19 age bracket, a linear association was observed in both unadjusted and adjusted models (Unadjusted: *P*_nonlinear = 0.419; Adjusted: *P *= 0.788). Conversely, in children aged 6–11, a significant nonlinearity emerged (*P*_nonlinear = 0.047), which became more pronounced post-adjustment for covariates (*P*_nonlinear = 0.022).

For a clearer presentation of odds ratios (OR) across quartiles of dietary protein intake, we stratified the intake into four quartiles and calculated the median intake of each. Comparing the risk at the 87.5th, 62.5th, and 37.5th percentiles with that at the 12.5th percentile, we found that the highest quartile's OR for overweight/obesity in the 6–19 age range was 1.64 (95% CI: 1.18–2.28, *P *= 0.004), and for those aged 6–11, it was 2.08 (95% CI: 1.40–3.08, *P *< 0.001). Even after adjusting for relevant covariates, the positive association persisted in both the 6–19 age group (OR = 1.61, 95% CI: 1.15–2.25, *P *= 0.006) and the 6–11 age group (OR = 2.07, 95% CI: 1.35–3.16, *P *= 0.001). However, no significant association was found between dietary protein intake and overweight/obesity in adolescents aged 12–19 across all models. Interestingly, the OR at the third quartile (Q3) was lower than those at the second (Q2) and fourth (Q4) quartiles, with none achieving statistical significance (*P *> 0.05) (Table 4).

**Table 4:** Logistic analysis of dietary protein intake and overweight/obesity in children and adolescents in the US, NHANES 2011–2020.

Age (years)	Quartile of estimated dietary protein intake							*P*_trend^e^
	Q1	Q2		Q3		Q4		
		OR (95% CI)	*P*	OR (95% CI)	*P*	OR (95% CI)	*P*	
6–19 (*n*^d^^ ^= 4,336)	1,279	1,213		1,089		755		
Unadjusted	Ref	1.43 (1.01, 2.03)	0.042	1.17 (0.88, 1.55)	0.300	1.64 (1.18, 2.28)	0.004	0.011
Model1^a^	Ref	1.40 (0.99, 1.98)	0.057	1.09 (0.81, 1.47)	0.600	1.53 (1.10, 2.14)	0.012	0.039
Model2^b^	Ref	1.47 (1.05, 2.05)	0.026	1.12 (0.83, 1.50)	0.400	1.58 (1.14, 2.19)	0.007	0.029
Model3^c^	Ref	1.47 (1.05,2 .05)	0.025	1.12 (0.83, 1.52)	0.400	1.61 (1.15, 2.25)	0.006	0.023
6–11 (*n*^d^^ ^= 2,927)	901	853		746		427		
Unadjusted	Ref	1.65 (1.04, 2.60)	0.033	1.28 (0.87, 1.88)	0.200	2.08 (1.40, 3.08)	<0.001	0.002
Model1^a^	Ref	1.60 (1.01, 2.54)	0.045	1.17 (0.79, 1.72)	0.400	1.90 (1.27, 2.86)	0.002	0.011
Model2^b^	Ref	1.67 (1.06, 2.65)	0.029	1.23 (0.83, 1.84)	0.300	2.06 (1.35, 3.14)	0.001	0.005
Model3^c^	Ref	1.66 (1.04, 2.64)	0.033	1.23 (0.82, 1.84)	0.300	2.07 (1.35, 3.16)	0.001	0.005
12–19(*n*^d^^ ^= 1,409)	374	358		341		327		
Unadjusted	Ref	1.09 (0.62, 1.93)	0.800	0.97 (0.62, 1.53)	0.900	1.02 (0.62, 1.66)	>0.900	>0.900
Model1^a^	Ref	1.13 (0.62, 2.06)	0.700	1.03 (0.67, 1.56)	>0.900	1.04 (0.63, 1.71)	0.900	>0.900
Model2^b^	Ref	1.26 (0.73, 2.15)	0.400	1.02 (0.67, 1.55)	>0.900	0.98 (0.60, 1.59)	>0.900	0.700
Model3^c^	Ref	1.30 (0.77, 2.20)	0.300	1.06 (0.69, 1.62)	0.800	1.03 (0.63, 1.68)	>0.900	>0.900

US, United States; NHANES, National Health and Nutrition Survey; OR, odds ratio; 95% CI: 95% Confidence Interval.

^a^ Model 1, adjusted for age, gender, ethnicity and household income-to-poverty ratio (PIR).

^b^ Model 2, adjusted for covariates of model 1 plus total energy intake and dietary fiber intake.

^c^ Model 3, according to model 2 plus the physical activity level.

^d^ *n*, number of samples.

^e^ *P*_ trend based on variable containing median value for each quintile.

According to the macronutrient substitution model (Table 5), substituting carbohydrate intake (5%E) for protein significantly reduced the risk of overweight/obesity in children aged 6–11 (*P*_trend = 0.016). Similarly, a consistent decrease in risk was noted when fat intake (5%E) replaced protein (*P*_trend < 0.001).

**Table 5:** The adjusted OR and 95%CI for the substitution of dietary carbohydrates and dietary fats with dietary protein intake in children and adolescents with overweight and obesity.

Age (years)	Quartile of estimated dietary protein intake							*P*_trend^b^
	Q1	Q2		Q3		Q4		
		OR (95% CI)	*P*	OR (95% CI)	*P*	OR (95% CI)	*P*	
6–19 (*n*^c^^ ^= 4,336)	1,279	1,213		1,089		755		
5E% higher carbohydrate and lower protein intake^a^	Ref	0.87 (0.56, 1.34)	0.5	0.64 (0.40, 1.00)	0.05	0.65 (0.40, 1.04)	0.07	0.025
5E% higher Fat and lower protein intake^a^	Ref	1.47 (1.05, 2.05)	0.025	1.12 (0.83, 1.52)	0.4	1.61 (1.15, 2.25)	0.006	<0.001
6–11 (*n*^c^^ ^= 2,927)	901	853		746		427		
5E% higher carbohydrate and lower protein intake^a^	Ref	0.83 (0.54, 1.27)	0.4	0.52 (0.33, 0.82)	0.005	0.44 (0.25, 0.75)	0.003	0.016
5E% higher Fat and lower protein intake^a^	Ref	1.09 (0.76, 1.57)	0.6	1.02 (0.74, 1.39)	>0.9	1.49 (1.10, 2.03)	0.012	<0.001

OR, odds ratio; 95% CI: 95% Confidence Interval.

^a^ The model was adjusted for age, gender, ethnicity, household income-to-poverty ratio (PIR), total energy intake, dietary fiber intake and the physical activity level.

^b^ *P*_ trend based on variable containing median value for each quintile.

^c^ *n*, number of samples.

Sensitivity analyses, after excluding potential false positives, corroborated these findings (Supplementary Table S1).

## 4 Discussion

This investigation utilized data from the NHANES 2011-March 2020, a nationally representative survey in the United States, to examine the association between dietary protein consumption and the prevalence of overweight and obesity in children and adolescents.

Nutritional intake is crucial for the physical development and metabolic health of this young demographic. Within this framework, protein is deemed an essential element of a well-rounded diet. High-protein diets, such as the ketogenic diet, have been reputed to promote rapid weight loss and have gained increasing recognition (31, 32). The standard dietary guidelines suggest that proteins should constitute approximately 10% of daily caloric intake, with an acceptable range of 10%–35% (33). Yet, our findings indicate that protein consumption within our study cohort notably surpasses these suggested levels (21, 34), prompting concerns regarding the potential negative metabolic implications of excessive protein consumption on energy balance. Prolonged high protein intake has been epidemiologically linked to elevated risks of obesity, diabetes, cardiovascular disease, and increased mortality (35). Our research identified a positive correlation between elevated dietary protein intake and heightened weight and BMI among children aged 6–11, while such an association was absent in adolescents aged 12–19. This observation is buttressed by several other studies, which have reported that increased protein consumption in early life is associated with greater BMI in later stages (36, 37). Moreover, results from a randomized clinical trial have demonstrated that high-protein infant formula intake in the first year of life leads to greater body weight at age two, as well as increased BMI and obesity risk before reaching six years of age (38). The evidence linking animal protein intake with obesity further corroborates our conclusions (39). In our study, we utilized BMI and weight status categorized based on BMI as outcome variables, which helped us better identify and predict the future risk of cardiovascular disease in children and adolescents with higher BMI. Since several studies have reported a strong association between elevated BMI during childhood or adolescence (even within the normal but higher range) and the later occurrence of CVD (8, 40). Our study also found that children from families with lower income poverty also showed a higher prevalence of overweight/obesity, whereas this association was absent in adolescents aged 12–19 years. This observation is supported by the study of Luis A. Rodríguez et al., which shows that children from poorer households with lower incomes consume healthier fatty acids with increasing age (41).

Additionally, we discerned a nonlinear relationship between protein intake and the incidence of overweight/obesity in children and adolescents via restricted cubic spline functions. For the cohort aged 6–11, an optimal median dietary protein intake-constituting 14%–15% of total energy consumption-negated the link with overweight/obesity (OR = 1.00). Yet, deviations toward lower or higher protein consumption correlated positively with an elevated risk of overweight/obesity. Correspondingly, logistic regression models categorized into quartiles revealed that the odds ratio for protein consumption in the third quartile (Q3) was more favorable than that in both the lowest quartile (Q1) and the remaining quartiles. This implies that a moderate dietary protein level does not influence the likelihood of obesity in children. Our hypothesis gained further support from alternative analytical models. Diminishing protein intake by 5% of total energy and substituting it with an isocaloric proportion of carbohydrates or fats attenuated or nullified the positive association with overweight/obesity risk. This aligns with Trudy et al.'s findings (21), which observed that protein intake-when offset by a corresponding decrease in fat or carbohydrate consumption-maintained a similar positive relationship with childhood BMI. These findings indicate that dietary protein management may be as critical in addressing overweight and obesity as the regulation of carbohydrates and fats. Recent clinical research has illustrated that a diet low in protein can augment metabolic health, foster weight loss, decrease obesity prevalence, and improve glycemic control, an observation echoed in animal studies (42). Further underscoring the advantages of a low-protein diet, the work of Dudley et al. (43) highlights its role in promoting stable glycemia and healthy body mass. Hence, in light of our results and the body of existing research, the formulation of evidence-based, age-specific dietary guidelines and public health initiatives becomes imperative. Such strategies should encourage suitable protein intake levels through dietary education and related interventions to prevent and ameliorate childhood obesity. Nevertheless, the specific role of dietary protein in overweight and obesity-particularly the impact of protein from varied sources and qualities on metabolic health-necessitates additional rigorous research and elucidation.

Research indicates that low-carbohydrate diets may be implicated in the onset of obesity (44), corroborating our observations that individuals, both children aged 6–11 and adolescents aged 12–19, who are overweight or obese generally consume fewer carbohydrates. Furthermore, higher fat intake among obese youths concurs with findings from other studies (45, 46), suggesting a complex interplay of consistent nutrient intake on the prevalence of obesity in younger populations. The established link between increased protein consumption and BMI might be due to the body's ability to convert excess protein into glucose via gluconeogenesis, which can then be stored as adipose tissue (47). The “early protein hypothesis” (48) further suggests that protein consumption exceeding the body's needs can promote insulin and insulin-like growth factor-1 (IGF1) secretion, accelerating growth and adipogenesis, while simultaneously inhibiting lipolysis (49). Evidently, forthcoming studies are tasked with further clarifying the role and underlying mechanisms of dietary protein, including its amino acid components, in the development and progression of overweight and obesity among children and adolescents.

This study has several limitations: First, the cross-sectional design precludes establishing causality. Secondly, reliance on the 24-h dietary recall method raises questions regarding its ability to represent the participants' habitual dietary patterns. Moreover, the lack of a significant relationship between dietary protein intake and obesity in adolescents aged 12–19 could stem from the reduced sample size within this demographic, coupled with the significant variability inherent to the transition from adolescence to adulthood, which complicates the accurate characterization of this group's attributes using available data. Lastly, while we observed varied prevalence rates of overweight and obesity among children and adolescents, with noticeable racial disparities echoing prior research (4, 28), our analysis did not extend to stratified examinations or discussions about different racial groups. Additionally, our results do not account for the potential interactive effects of socioeconomic status on the correlation between dietary protein intake and the incidence of overweight and obesity in the young population. We noted that children from middle to high-income families had a lower propensity for obesity than their counterparts from lower-income households. Recent studies suggest that children of lower socioeconomic standing may face environments conducive to obesity (50, 51), where economic constraints restrict access to nutritious food (52), leading to the consumption of inferior protein or nutritionally deficient diets (53). In addition, the NHANES study lacked a 24-h urine sample to measure protein, so it is suggested that subsequent studies should consider the combination of urine protein monitoring and dietary survey data to obtain more accurate results.

## 5 Conclusions

In conclusion, our findings indicate that in U.S. children aged 6–11 years, a higher dietary protein intake could be correlated with an increased risk of obesity. However, this association was not observed in U.S. adolescents aged 12–19 years. Further research, especially longitudinal studies employing 24-h urine collection to assess protein intake, is necessary to elucidate the potential causal relationships between protein types and obesity.

## Data Availability

The datasets presented in this study can be found in online repositories. The names of the repository/repositories and accession number(s) can be found in the article/Supplementary Material.
