# Supplementary material for: Association between protein-to-energy ratio and overweight/obesity in children and adolescents in the United States: a cross-sectional study based on NHANES
Source: Front Pediatr. 2024 Jun 25;12:1383602. doi: 10.3389/fped.2024.1383602 (PMC11232357; doi:10.3389/fped.2024.1383602)
Supplement: Supplementary file 1 [file Table1.docx]

**Supplementary Material**

**sTable1.** Logistic Analysis of Dietary Protein Intake and Obesity in Children and Adolescents in the US,NHANES 2011-2020

|  | **Q1** | **Q2** | | **Q3** | | **Q4** | |  |
| --- | --- | --- | --- | --- | --- | --- | --- | --- |
|  |  | **OR(95%CI)^d^** | ***P*** | **OR(95%CI)^d^** | ***P*** | **OR(95%CI)^d^** | ***P*** | ***P_*trend^e^** |
| **Aged 6-11 years** | 469 | 516 |  | 455 |  | 395 |  |  |
| Unadjusted | Ref | 1.72(1.02,2.89) | 0.042 | 1.27(0.81,1.99) | 0.300 | 2.00(1.25,3.18) | 0.004 | 0.017 |
| Model1^a^ | Ref | 1.63(0.98,2.72) | 0.061 | 1.12(0.70,1.80) | 0.600 | 1.76(1.08,2.86) | 0.025 | 0.091 |
| Model2^b^ | Ref | 1.76(1.02,3.04) | 0.041 | 1.24(0.75,2.05) | 0.400 | 2.00(1.17,3.42) | 0.012 | 0.044 |
| Model3^c^ | Ref | 1.78(1.02,3.09) | 0.042 | 1.23(0.74,2.05) | 0.400 | 2.04(1.19,3.48) | 0.010 | 0.037 |
| **Aged 12-19 years** | 150 | 126 |  | 120 |  | 94 |  |  |
| Unadjusted | Ref | 1.59(0.92,2.74) | 0.095 | 1.03(0.60,1.76) | >0.99 | 0.95(0.56,1.62) | 0.900 | 0.800 |
| Model1^a^ | Ref | 1.63(0.90,2.95) | 0.100 | 1.05(0.61,1.81) | 0.900 | 0.94(0.56,1.58) | 0.800 | >0.900 |
| Model2^b^ | Ref | 1.64(0.90,2.98) | 0.110 | 1.06(0.61,1.83) | 0.800 | 0.95(0.56,1.62) | 0.800 | 0.300 |
| Model3^c^ | Ref | 1.75(0.90,3.40) | 0.100 | 0.98(0.56,1.70) | >0.99 | 1.05(0.57,1.93) | 0.900 | >0.900 |

US: United States; NHANES: National Health and Nutrition Survey; OR: odds ratio; 95%CI: 95% Confidence Interval.

^a^Model 1, adjusted for age, gender, ethnicity and household income-to-poverty ratio (PIR).

^b^Model 2, adjusted for covariates of model 1 plus total energy intake and dietary fiber intake;

^c^Model 3, according to model 2 plus the physical activity level;

^e^*P*_ trend based on variable containing median value for each quintile.
